# Quality of life after wearing multifocal contact lenses for myopia control for 2 weeks in the BLINK Study

**DOI:** 10.1111/opo.13216

**Published:** 2023-08-23

**Authors:** Anita Ticak, Jeffrey J. Walline, David A. Berntsen, Donald O. Mutti, Lisa A. Jones-Jordan, Laura Cardenas, Elizabeth Day, Bradley E. Dougherty, The BLINK Study Group, Donald F. Everett, Jimmy Le, Kimberly J. Shaw, Jenny Huang Jones, Loraine T. Sinnott, Krystal L. Schulle, Dashaini V. Retnasothie, Amber Gaume Giannoni, Maria K. Walker, Moriah A. Chandler, Mylan Nguyen, Lea Hair, Augustine N. Nti, Jill A. Myers, Alex D. Nixon, Katherine M. Bickle, Gilbert E. Pierce, Kathleen S. Reuter, Dustin J. Gardner, Andrew D. Pucker, Matthew Kowalski, Ann Morrison, Danielle J. Orr, Janet T. Holbrook, Jane Gwiazda, Timothy B. Edrington, John Mark Jackson, Charlotte E. Joslin

**Affiliations:** 1 University of Houston College of Optometry, Houston, Texas USA; 2 The Ohio State University College of Optometry, Columbus, Ohio USA

**Keywords:** contact lens, multifocal, myopia, paediatric, quality of life

## Abstract

**Purpose:**

To validate Pediatric Refractive Error Profile 2 (PREP2) subscales that can be used to evaluate contact lens wearers and compare vision-specific quality of life measurements between children wearing multifocal and single vision contact lenses for 2 weeks.

**Methods:**

Two hundred and ninety-four myopic children aged 7–11 years (inclusive) were enrolled in the 3-year, double-masked Bifocal Lenses In Nearsighted Kids (BLINK) Study. Participants completed the PREP2 survey after having worn contact lenses for 2 weeks. The Vision, Symptoms, Activities and Overall PREP2 subscales were used to compare participants' subjective assessment while wearing +1.50 or +2.50 D add multifocal or single vision contact lenses. Rasch analysis was used to validate each subscale and to compare participants' subjective assessment of contact lens wear.

**Results:**

Item fit to the Rasch model was good for all scales, with no individual items having infit mean square statistics outside the recommended range (0.7–1.3). Response category function was acceptable for all subscales, with ordered category thresholds. Measurement precision, assessed by the Rasch person reliability statistic, was less than ideal (≥0.8) for three of the subscales, but met the minimum acceptable standard of 0.5. Scores for the Vision subscale differed by treatment assignment (*p* = 0.03), indicating that participants with the highest add power reported statistically worse quality of vision, although the difference was only 3.9 units on a scale of 1–100. Girls reported fewer symptoms than boys (*p* = 0.006), but there were no other differences between boys and girls.

**Conclusions:**

Rasch analysis demonstrates that the PREP2 survey is a valid instrument for assessing refractive error-specific quality of life. These results suggest that vision-related quality of life is not meaningfully affected by 2 weeks of soft multifocal contact lens wear for myopia control.

## Key points

- Wearing +2.50 D add power contact lenses may have a trivial negative effect on the vision-related quality of life in children undergoing myopia control.
- Although children wearing +2.50 D add power contact lenses may report slightly lower quality of vision, they did not experience a significant difference in symptoms, activities or overall quality of life.
- The Pediatric Refractive Error Profile 2 provides a valid assessment of subjective issues related to soft multifocal contact lens myopia control.

## INTRODUCTION

Myopia is the most common ocular disorder worldwide, the leading cause of visual impairment in children and has an incidence that is increasing rapidly.^1,2^ In fact, the prevalence of myopia and high myopia (defined as < −5.00 dioptres [D]) are projected to affect five billion and one billion people by 2050, respectively.^1^ In the United States, the prevalence of myopia has increased from 25% in the 1970s to >33% currently.^3^ Furthermore, the risk of pathological complications such as glaucoma, cataract, retinal detachment and myopic maculopathy—as well as the economic impact and burden of healthcare associated with myopia—has become a worldwide issue.^4–6^ The World Health Organization stated that the estimated costs of the coverage gap for unaddressed refractive errors are US$16 billion.^7^ The negative health impact and financial burden of myopia progression globally has motivated researchers to focus on treatments to reduce the risk of myopia onset and progression.

Surveys can assess the quality of life and vision during treatments for myopia control. Vision-related quality-of-life surveys can help researchers determine whether a myopia treatment was beneficial, well-tolerated and whether visual quality changed over time. To date, vision-related quality-of-life surveys in refractive correction research have been used to assess a variety of treatment modalities including orthokeratology, multifocal soft contact lens designs and spectacles in adults.^8,9^ The Pediatric Refractive Error Profile (PREP) survey was created specifically to measure vision-related quality of life in paediatric populations. The content of the PREP survey was derived from focus groups of 8- to 14-year-old contact lens wearers, who were asked questions about contact lens wear. Their responses were transcribed and qualitatively assessed for content and quantitatively categorised to determine frequency. From that process, scales were created to cover all of the areas, and items were written in language used by children. The PREP survey was used in the Contact Lenses In Pediatrics^10^ and Adolescent and Child Health Initiative to Encourage Vision Empowerment studies.^11^ These studies demonstrated that single vision contact lens use significantly improved vision-related quality of life,^10^ as reported by children and teens using the PREP survey.^11^ A 2010 study used PREP to show that myopic children <12 years of age reported better vision-related quality of life when fitted with contact lenses compared to glasses. The findings also showed that children who are older, participate in recreational activities, are motivated to wear contact lenses and did not like their appearance in glasses would likely benefit most from contact lens use.^10^ However, one limitation of the PREP survey was poor repeatability, which limited the ability to compare participants over time.^10^

The PREP2 was developed with the intent to standardise the distribution of positively and negatively worded items, make subscales containing a similar number of questions for each construct and improve repeatability. The PREP2 contains seven subscales that cover a variety of constructs related to refractive error and its correction, including visual function, ocular symptoms (comfort, irritation, etc.), appearance (looks and effect of refractive correction), activities (sports and outdoor activities), peer perceptions, handling and opinion of refractive correction. One investigation concluded that the 56-item PREP2 survey showed adequate repeatability and validity with better repeatability on most subscales than the original PREP, and higher correlation between the first and second administration.^12^ The authors who developed PREP2 also suggested that further validation studies be conducted using Rasch analysis. In recent years, both the Contact Lens Impact on Quality of Life (CLIQ) and Quality of Vision (QoV) questionnaires have shown validity and repeatability using Rasch analysis.^13,14^

The purpose of the present analysis was to validate PREP2 subscales that can be used to compare contact lens wearers as well as vision-specific quality of life between children wearing multifocal and single vision contact lenses.

## METHODS

### Participants

Two hundred and ninety-four myopic children aged 7–11 years (inclusive) were enrolled in the Bifocal Lenses in Nearsighted Kids (BLINK) Study, a 3-year, double-masked randomised clinical trial at two sites (The University of Houston College of Optometry and the Ohio State University College of Optometry) from September 2014 to June 2016. Enrolled participants had best-corrected, high contrast, distance visual acuity of ≤+0.10 logMAR, spherical refractive error of −0.75 to −5.00 D and ≤1.00 D astigmatism in each eye, as well as ≤2.00 D anisometropia by cycloplegic autorefraction. Full details of baseline characteristics and methods were reported previously,^15^ but the details and methods relevant to this analysis are described below. The research adhered to the tenets of the Declaration of Helsinki, and parental/guardian permission and child assent were obtained. The institutional review boards at the University of Houston and the Ohio State University approved this research.

### Randomisation

Participants were randomly assigned to wear single vision Biofinity, Biofinity multifocal D with a +1.50 add, or Biofinity multifocal D with a +2.50 add contact lenses (coopervision.com) in both eyes. The randomisation was stratified by clinical site and age groups (7–9 vs. 10–11 years of age) using a random permuted block design to ensure sequential balance of the three treatment groups and to prevent knowledge of subsequent treatment assignment.

### Contact lenses

The silicone-hydrogel contact lenses used in the study were made of comfilcon A (48% water), had an 8.6 mm base curve and an overall diameter of 14.0 mm. The ‘D’ multifocal lens design incorporates a spherical central distance zone of approximately 3 mm in diameter, surrounded by a zone of progressively increasing plus power before achieving full add power at the outer zone.^16–18^

### Wearing time

Wearing time after 2 weeks of wear was assessed by asking parents the number of weekdays and weekend days that the participant wore contact lenses and the typical time they inserted and removed the lenses during those periods. After calculating the time between insertion and removal on both weekdays and weekend days, the number of hours was multiplied by the number of days. The total number of hours over the week was then divided by seven to calculate the average number of hours that contact lenses were worn per day.

### PREP2

The PREP2 consisted of seven subscales and 56 total items: Vision, Symptoms, Appearance, Activities, Handling, Peer Perceptions and Overall. The Overall subscale was a separate scale that measured the overall quality of life, not an average score of other subscales. Each scale contained four positively phrased and four negatively phrased items (see Appendix 1). Based on the content of the items, four of the PREP2 subscales were deemed appropriate to compare contact lens wearers in this study: Vision, Symptoms, Activities and Overall. The remaining three subscales targeted differences in perception, appearance and ease of handling between spectacles and contact lenses, and since all of the participants in this study only wore contact lenses, these subscales were not analysed.

Participants completed the PREP2 survey after the initial 2 weeks of lens wear using REDCap, a web-based electronic data capture platform (project-redcap.org).^19^ While the PREP2 survey was completed by participants every 6 months for 3 years, here we report only findings from the initial 2 weeks of wear in order to establish the validity of the survey. Subsequent investigations will examine longitudinal changes in vision-specific quality of life. Parents were not allowed to help their child answer the questions. If the child had difficulty completing the survey, the examiner or co-ordinator read the survey to the participant. The examiner was allowed to answer questions about the meaning of a word but could not guide the participant to a specific answer.

### Survey scoring and analysis

Rasch analysis was used to generate summary scores (Rasch ‘person measures’) for each subscale and to assess the psychometric properties of the PREP2. The Rasch model is widely used for scoring and evaluating surveys in the health sciences. It allows for interval-level scoring from the ordinal-level responses and valid analysis of the scores with parametric statistics.^20^ Person measures are expressed in logits, with higher values signifying more of the construct being measured (e.g., visual function or symptoms). For ease of interpretation, the logit scores were converted to a 0 (poor quality of life) to 100 (excellent quality of life) scale.

The measurement properties of the survey were evaluated using published guidelines.^21,22^ The fit of each survey item to the Rasch model was assessed using the infit mean square statistic. Items with infit mean square values >1.4 were removed iteratively, beginning with the most misfitting item, until all values were acceptable. The measurement precision of each subscale was assessed with the person separation index. Principal component analysis of model residuals was used to assess whether each subscale was unidimensional, a key assumption of the Rasch model. Response category functioning was assessed using plots of the response probability curves, checking to ensure that the probability curves were ordered and that no response category was underused. More detailed summaries of the use of Rasch analysis for survey evaluation are available elsewhere.^21,23,24^

### Cycloplegic refractive error

Refractive error was measured by cycloplegic autorefraction using the Grand Seiko WAM-5500 autorefractor (grandseiko.com). After instilling one drop of 0.5% proparacaine or tetracaine, cycloplegia was achieved by instilling two drops of 1.0% tropicamide, separated by 5 min. Measurements were performed 25 min after the second drop of tropicamide. Ten spherocylindrical autorefraction measurements were obtained while the participant fixated 0.18 logMAR (6/9) size letters on a near point test card viewed through a +4.00 D Badal lens. The letters were presented at optical infinity, and then moved to a slightly blurred position to ensure relaxation of residual accommodation.^25^ The 10 spherocylindrical autorefractions were averaged using the power vector analysis described by Thibos et al.^26^

### Statistical analysis

Winsteps version 4.5.1 (winsteps.com) was used to perform the Rasch analysis using the Andrich rating scale model.^27,28^ SPSS Statistics version 28 (ibm.com) was used for statistical testing. ANOVA tests and Spearman's correlations were used to assess the relationships among scores on the PREP2 subscales, participant characteristics and contact lens add power.

## RESULTS

### Participant characteristics

Two hundred and eighty-nine participants completed the survey 2 weeks after receiving the contact lenses. The mean (SD) age at enrolment was 10 (1) years (range = 7–11 years) and 60.2% of participants was female. A majority (68.5%) of participants were white, 10.4% reported more than one race, 9.7% were black and 8.3% were Asian. Seventy-seven (28.6%) participants identified as Hispanic/Latino. Mean (SD) baseline cycloplegic spherical equivalent autorefraction of the right eye for all participants was −2.38 (1.01 D) (range = −0.82 to −5.49 D). There were no significant differences in pupil size among treatment groups (mesopic, *p* = 0.66; photopic, *p* = 0.51). The mean (± SD) mesopic and photopic pupil size across treatment groups over 3 years were 6.4 ± 0.7 mm (range 4.5–8.4 mm) and 5.0 ± 0.6 mm (range: 3.3–6.9 mm), respectively. Participants wore their contact lenses 10.3 + 2.8 hours per day, and there was no difference between the treatment groups (one-way ANOVA, *p* = 0.50).

### Survey properties

Results of the Rasch analysis of the various PREP2 subscales are reported in Table 1. Response category function, not shown in Table 1, was acceptable for all subscales, with ordered category thresholds. Item reliability (≥0.8 is ideal) was acceptable for all subscales. Item fit to the Rasch model was good for all scales, with no individual items having infit mean square statistics outside the recommended range of 0.7–1.3. Measurement precision, assessed by the Rasch person reliability statistic, was less than ideal (≥0.8) for three of the subscales, but all met the minimum acceptable standard of 0.5.^21,24^ The Symptoms subscale demonstrated the best measurement precision. There was no evidence of multidimensionality by principal component analysis of model residuals for any of the scales, with the eigenvalue of the first contrast of the principal component analysis below 2.0 for all four subscales.

**TABLE 1:** Results of the Rasch analysis of the PREP2 subscales used for this analysis.

Subscale	SE of item mean	Item reliability	Mean item infit mean square	Mean Rasch person measure (SE)	Person reliability
Vision	2.07	0.98	1.03	61.00 (0.67)	0.77
Symptoms	0.76	0.88	0.99	60.92 (0.79)	0.84
Activities	1.96	0.97	1.01	64.74 (0.96)	0.72
Overall	2.68	0.98	1.03	67.37 (0.78)	0.70

Abbreviations: PREP2, Pediatric Refractive Error Profile 2; SE, Standard error.

### Contact lens treatment assignment, participant characteristics and PREP2 scores

**FIGURE 1:**
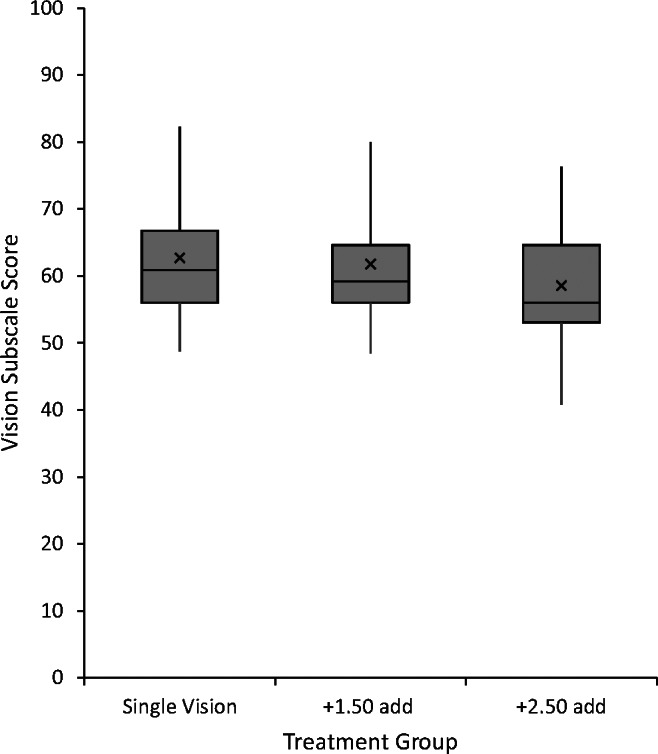
Boxplot of PREP2 Vision subscale scores (0 = poor quality of life and 100 = excellent quality of life) by treatment group. The centre line indicates the median score, the extent of the boxes indicates the 25th and 75th percentiles, the whiskers indicate the 5th and 95th percentiles and the ‘x’ indicates the mean.

Scores for the Vision subscale differed by treatment assignment (*p* = 0.03), indicating that participants with the highest add power reported statistically worse visual functioning than single vision contact lens wearers (Tukey HSD, *p* = 0.03). There was no significant difference between the +1.50 add and single vision nor between the +1.50 and +2.50 add powers (Figure 1).

There were no significant differences among treatment groups for the other PREP2 subscale scores (Table 2).

**TABLE 2:** Mean Pediatric Refractive Error Profile 2 mean (SD) subscale scores (0 = poor quality of life and 100 = excellent quality of life) by treatment group, sex and age group (7–9 vs. 10–11 years).

	Vision	Symptoms	Activities	Overall
Treatment group				
Single vision	62.7 (11.2)	60.7 (12.6)	63.9 (15.4)	67.3 (12.6)
+1.50 add	61.8 (10.8)	61.3 (12.6)	67.0 (17.9)	68.2 (12.9)
+2.50 add	58.6 (11.8)	60.8 (15.2)	63.3 (15.2)	66.7 (14.1)
*p*-Value	0.03	0.95	0.23	0.73
Sex				
Girls	61.4 (11.5)	62.7 (14.6)	65.7 (16.3)	68.2 (12.7)
Boys	60.3 (11.1)	58.3 (11.2)	63.3 (16.1)	66.0 (13.8)
*p*-Value	0.41	0.01	0.22	0.17
Age (years)				
7–9	60.0 (11.3)	60.0 (14.0)	63.9 (16.7)	65.3 (12.5)
10–11	61.7 (11.4)	61.5 (13.2)	65.3 (16.0)	68.7 (13.5)
*p*-value	0.23	0.36	0.46	0.03
Baseline myopia (cycloplegic spherical equivalent, right eye)				
Not more than 2.13 D of myopia	60.6 (11.5)	60.6 (13.4)	64.0 (16.2)	67.6 (14.0)
More than 2.13 D of myopia	61.4 (11.2)	61.3 (13.5)	65.4 (16.4)	67.1 (12.3)
*p*-Value	0.56	0.65	0.48	0.73

Girls reported fewer symptoms than boys on the Symptoms subscale (*p* = 0.006), but there were no other statistically significant differences between boys and girls on the other PREP2 subscales. Also, the older group reported better Overall subscale scores than the younger group (Student's *t*-test, *p* = 0.03), but there were no other statistically significant differences by age group. There was also no significant difference for any of the scales between participants with not more than the baseline median cycloplegic spherical equivalent myopia in the right eye compared with those with more than the median value (Table 2).

## DISCUSSION

BLINK Study participants who wore the +2.50 add contact lenses reported statistically worse but clinically similar quality of vision on the PREP2 Vision subscale compared with the children wearing single vision or +1.50 add contact lenses. This finding suggests that high add power contact lenses may have a negligible effect on vision-related quality of life in children being treated for myopia control, similar to the statistically worse low contrast visual acuity for multifocal contact lens wearers than single vision contact lens wearers, where the difference is only a couple of letters.^29^

There is evidence that other aspects of vision, such as contrast sensitivity,^30–32^ higher order aberrations,^30^ glare,^30^ reading speed^33^ and subjective vision ratings,^9^ may be negatively affected by multifocal lens wear. These reports highlight the effects that a multifocal design has on vision beyond high contrast visual acuity, which could be responsible for the differing visual experience between groups in our study. We did not find any relationship between contact lens add power and the PREP2 scales that measure symptoms, activities or overall opinion of the contact lenses. These findings suggest that, though they may report slightly lower quality of vision, in many respects, the children assigned higher add contact lenses did not experience much difference in their vision-related quality of life.

On average, girls reported lower levels of symptoms (higher score on the Symptoms subscale) than boys, and to our knowledge this has not been documented in other studies of children being treated for myopia progression. One investigation of adults reported that comfort upon insertion of contact lenses was better in females than in males, which would agree with our findings, but there was no difference in comfort reported by the sexes during or at the end of the day.^34^ While the difference between boys and girls was statistically significant, a difference of four points on a 100-point scale is unlikely to be dramatic, which may explain why the study of adults reported better comfort for women upon insertion, but not during the day or at the end of the day.

A comparison between higher and lower myopes did not provide any significant differences in any of the subscales. This is not surprising because all participants were required to have 0.10 logMAR (6/7.5) or better best-corrected visual acuity at baseline, so we did not expect a difference in vision or quality of life based on the amount of baseline myopia.

The Symptoms subscale showed the best measurement precision (Rasch person separation) of the PREP2 scales examined, and it may make a good short form measurement instrument for the assessment of symptoms in children wearing contact lenses. One currently available option for the measurement of symptoms in contact lens wearers is the 28-item CLIQ Questionnaire. Rasch analysis and standard psychometric analyses demonstrated that this is a valid and reliable measure of quality of life in contact lens wearers.^13^ The survey was developed for the pre-presbyopic wearer but, to date, has not had widespread use in the literature as a symptom survey for paediatric studies. Overall, there seems to be a gap regarding survey use in children. Measuring a participant's vision-related quality of life is a growing metric in myopia management research, which highlights numerous possible influences on a patient's vision-related quality of life. Additional knowledge in this area may help guide future myopia management considerations.^35^ Currently, there seems to be evidence supporting the need for validated and reliable measures to assess and drive decision-making in myopia management. The use of the PREP2 Symptoms subscale itself may be a beneficial and concise tool to use in future studies of myopia management in young populations.

One limitation of this study is that measurement precision, as assessed by the Rasch person reliability statistic, was less than the published ideal standards for the Activities, Overall and Vision subscales of the PREP2 (although minimum standards for a useful measurement were met).^23,24^ This could have limited our ability to detect differences between groups and associations with participant characteristics. Future studies may require more participants to validate the survey further. Another limitation is the geographical location of the study which possibly under-represented differences in culture, environment and race/ethnicity.

Rasch analysis showed that the PREP2 provides a valid assessment of subjective issues related to soft multifocal contact lens myopia control. The only difference detected between treatment groups indicated that soft multifocal contact lenses may affect vision, but the effect is minor. All other subscales showed no significant difference between treatment groups. These findings suggest that multifocal contact lenses, shown to slow myopia progression and eye growth effectively,^29^ provide very little compromise in children's subjective assessment of vision, comfort and activities, thus providing a safe and effective means of myopia control.

## APPENDIX 2

### BLINK Study Group

*Executive Committee*: Jeffrey J. Walline (Study Chair, The Ohio State University College of Optometry), David A. Berntsen (UH Clinic Principal Investigator, University of Houston College of Optometry), Donald O. Mutti (OSU Clinic Principal Investigator, The Ohio State University College of Optometry), Lisa A. Jones-Jordan (Data Coordinating Center Director, The Ohio State University College of Optometry), Donald F. Everett (NEI Program Official), Jimmy Le (NEI Program Official, 2019–present).

*Chair's Centre*: Kimberly J. Shaw (Study Coordinator, The Ohio State University College of Optometry), Jenny Huang Jones (Investigator, The Ohio State University College of Optometry), Bradley E. Dougherty (Survey Consultant, The Ohio State University College of Optometry).

*Data Coordinating Centre*: Loraine T. Sinnott (Biostatistician, The Ohio State University College of Optometry).

*University of Houston Clinic Site (University of Houston College of Optometry)*: Laura Cardenas (Clinic Coordinator), Krystal L. Schulle (Unmasked Examiner, 2014–2019), Dashaini V. Retnasothie (Unmasked Examiner, 2014–2015), Amber Gaume Giannoni (Masked Examiner), Anita Tićak (Masked Examiner), Maria K. Walker (Masked Examiner), Moriah A. Chandler (Unmasked Examiner, 2016–present), Mylan Nguyen (Data Entry, 2016–2017), Lea Hair (Data Entry, 2017–2019), Augustine N. Nti (Data Entry, 2019–present).

*Ohio State University Clinic Site (The Ohio State University College of Optometry)*: Jill A. Myers (Clinic Coordinator), Alex D. Nixon (Unmasked Examiner, 2014–2019), Katherine M. Bickle (Unmasked Examiner), Gilbert E. Pierce (Unmasked Examiner, 2014–2019, deceased), Kathleen S. Reuter (Masked Examiner, 2014–2019), Dustin J. Gardner (Masked Examiner, 2014–2016), Andrew D. Pucker (Masked Examiner, 2015–2016), Matthew Kowalski (Masked Examiner, 2016–2017), Ann Morrison (Masked Examiner, 2017–2019), Danielle J. Orr (Unmasked Examiner, 2018–present), Elizabeth Day (Unmasked Examiner, 2019–present).

*Data Safety and Monitoring Committee*: Janet T. Holbrook (Chair, Johns Hopkins Bloomberg School of Public Health), Jane Gwiazda (Member, New England College of Optometry), Timothy B. Edrington (Member, Southern California College of Optometry at Marshall B. Ketchum University), John Mark Jackson (Member, Southern College of Optometry), Charlotte E. Joslin (Member, University of Illinois at Chicago).

## APPENDIX 1

### Prep2 survey

**Table:**

1.	My vision is very clear when I look far away (movies or board at school)	Strongly disagree	Disagree	Neutral	Agree	Strongly agree
2.	My eyes are sometimes uncomfortable	Strongly disagree	Disagree	Neutral	Agree	Strongly agree
3.	I am happy with the way that I look	Strongly disagree	Disagree	Neutral	Agree	Strongly agree
4.	When I play sports or other activities, I sometimes don't wear vision correction because it bothers me	Strongly disagree	Disagree	Neutral	Agree	Strongly agree
5.	When I play outdoors, I never have a problem with my vision correction	Strongly disagree	Disagree	Neutral	Agree	Strongly agree
6.	My friends make fun of me because of my vision correction	Strongly disagree	Disagree	Neutral	Agree	Strongly agree
7.	I love my vision correction	Strongly disagree	Disagree	Neutral	Agree	Strongly agree
8.	When I look far away, my vision is not as clear as I would like it to be	Strongly disagree	Disagree	Neutral	Agree	Strongly agree
9.	My eyes are always comfortable	Strongly disagree	Disagree	Neutral	Agree	Strongly agree
10.	I do not like how I look when I wear my vision correction	Strongly disagree	Disagree	Neutral	Agree	Strongly agree
11.	When I play outdoors, I never have a problem with my vision correction	Strongly disagree	Disagree	Neutral	Agree	Strongly agree
12.	My vision correction sometimes breaks or falls off while I am wearing it	Strongly disagree	Disagree	Neutral	Agree	Strongly agree
13.	My friends want the same kind of vision correction that I have	Strongly disagree	Disagree	Neutral	Agree	Strongly agree
14.	I don't like my vision correction very much	Strongly disagree	Disagree	Neutral	Agree	Strongly agree
15.	My vision is very clear when I look at something close (books or cell phones)	Strongly disagree	Disagree	Neutral	Agree	Strongly agree
16.	My eyes sometimes itch, burn, or feel dry	Strongly disagree	Disagree	Neutral	Agree	Strongly agree
17.	My vision correction makes me look cool	Strongly disagree	Disagree	Neutral	Agree	Strongly agree
18.	When I play outside, my vision correction sometimes bothers me	Strongly disagree	Disagree	Neutral	Agree	Strongly agree
19.	When I am active, my vision correction never falls off	Strongly disagree	Disagree	Neutral	Agree	Strongly agree
20.	My friends don't like how I look when I wear my vision correction	Strongly disagree	Disagree	Neutral	Agree	Strongly agree
21.	I never have problems with my vision correction	Strongly disagree	Disagree	Neutral	Agree	Strongly agree
22.	When I read, my vision is not as clear as I would like it to be	Strongly disagree	Disagree	Neutral	Agree	Strongly agree
23.	My eyes never feel irritated	Strongly disagree	Disagree	Neutral	Agree	Strongly agree
24.	I think that I could be better looking	Strongly disagree	Disagree	Neutral	Agree	Strongly agree
25.	I am never bothered by my vision correction when I am active (sports, dance, etc.)	Strongly disagree	Disagree	Neutral	Agree	Strongly agree
26.	My vision correction is sometimes hard to put on or take off	Strongly disagree	Disagree	Neutral	Agree	Strongly agree
27.	My friends only say good things about my vision correction	Strongly disagree	Disagree	Neutral	Agree	Strongly agree
28.	I wish I had a different kind of vision correction	Strongly disagree	Disagree	Neutral	Agree	Strongly agree
29.	My vision is always excellent	Strongly disagree	Disagree	Neutral	Agree	Strongly agree
30.	I am sometimes uncomfortable when I wear my vision correction	Strongly disagree	Disagree	Neutral	Agree	Strongly agree
31.	When I wear my vision correction, I like how I look	Strongly disagree	Disagree	Neutral	Agree	Strongly agree
32.	I am worse at sports because my vision correction bothers me	Strongly disagree	Disagree	Neutral	Agree	Strongly agree
33.	My vision correction never gets lost or broken	Strongly disagree	Disagree	Neutral	Agree	Strongly agree
34.	My friends sometimes say things that are not nice about my vision correction	Strongly disagree	Disagree	Neutral	Agree	Strongly agree
35.	I like to wear my vision correction	Strongly disagree	Disagree	Neutral	Agree	Strongly agree
36.	Sometimes my vision is not clear	Strongly disagree	Disagree	Neutral	Agree	Strongly agree
37.	My eyes always feel great	Strongly disagree	Disagree	Neutral	Agree	Strongly agree
38.	When I look in the mirror, I do not like how I look	Strongly disagree	Disagree	Neutral	Agree	Strongly agree
39.	I never have any problems when I wear my vision correction while I play sports or do other activities	Strongly disagree	Disagree	Neutral	Agree	Strongly agree
40.	Sometimes it is hard to clean my vision correction	Strongly disagree	Disagree	Neutral	Agree	Strongly agree
41.	My friends never mention my vision correction	Strongly disagree	Disagree	Neutral	Agree	Strongly agree
42.	In general, wearing my vision correction bothers me	Strongly disagree	Disagree	Neutral	Agree	Strongly agree
43.	I can always see better than my friends	Strongly disagree	Disagree	Neutral	Agree	Strongly agree
44.	Sometimes I don't like how my eyes feel	Strongly disagree	Disagree	Neutral	Agree	Strongly agree
45.	Nobody notices when I wear my vision correction	Strongly disagree	Disagree	Neutral	Agree	Strongly agree
46.	I could be better at sports if I didn't have to wear vision correction	Strongly disagree	Disagree	Neutral	Agree	Strongly agree
47.	It is easy to put on or take off my vision correction	Strongly disagree	Disagree	Neutral	Agree	Strongly agree
48.	My friends sometimes laugh about my vision correction	Strongly disagree	Disagree	Neutral	Agree	Strongly agree
49.	I don't even notice my vision correction	Strongly disagree	Disagree	Neutral	Agree	Strongly agree
50.	My friends usually see better than me	Strongly disagree	Disagree	Neutral	Agree	Strongly agree
51.	Wearing my vision correction is always comfortable	Strongly disagree	Disagree	Neutral	Agree	Strongly agree
52.	Wearing my vision correction makes me look worse	Strongly disagree	Disagree	Neutral	Agree	Strongly agree
53.	I can play outside without ever thinking about my vision correction	Strongly disagree	Disagree	Neutral	Agree	Strongly agree
54.	I don't like cleaning my vision correction	Strongly disagree	Disagree	Neutral	Agree	Strongly agree
55.	When I wear my vision correction, my friends like the way I look	Strongly disagree	Disagree	Neutral	Agree	Strongly agree
56.	I hate wearing vision correction	Strongly disagree	Disagree	Neutral	Agree	Strongly agree
